# First record of *Scina nana* Wagler, 1926 (Crustacea, Amphipoda, Hyperiidea) in Mediterranean Sea from the northern coastal waters north of Syria (eastern Mediterranean)

**DOI:** 10.3897/BDJ.14.e199166

**Published:** 2026-07-16

**Authors:** Haidar Bassam Hasan, Adib Hasan Zeini, Wassim Mahmoud Mayya

**Affiliations:** 1 Latakia University, Latakia, Syria Latakia University Latakia Syria; 2 Damascus University, Damascus, Syria Damascus University Damascus Syria

**Keywords:** first record, Hyperiidea, Mediterranean, *Scina nana*, Syria

## Abstract

**Background:**

Global biodiversity repositories, including GBIF, OBIS and the authoritative taxonomic database WoRMS, consistently indicate that *Scina nana* is a globally rare holoplanktonic amphipod. None of these databases contains any historical or contemporary evidence of the species within the Mediterranean Basin. This complete absence of records strongly suggests that the specimen documented from Syrian coastal waters represents the first known occurrence of *S. nana* in the eastern Mediterranean.

**New information:**

In this study, *Scina nana* was observed for the first time in the coastal waters of Syria (Latakia Basin), this species being found mainly in tropical regions of all the world’s oceans, except the Mediterranean Sea. Given the complete lack of previous Mediterranean records, the appearance of *S. nana* in the region is most plausibly interpreted as a recent event, supporting its classification as non-indigenous species that has only recently entered the basin. The rapid warming of Mediterranean waters in recent decades may have provided conditions favourable for the arrival or temporary establishment of this species. Additional factors, such as broader changes in the thermohaline structure of the eastern Mediterranean, may also have facilitated its introduction to the Syrian coast.

## Introduction

Hyperiidean amphipods are important marine crustacean zooplanktons, ranking third in abundance after Copepoda and Euphausiacea (Shih 1982). Some species can be found in cold waters, where they constitute an important food source for marine mammals, sea birds and fish (Lavaniegos and Ohman 1999).

The family Scinidae (Stebbing 1888) comprises small to medium-sized Crustaceans, ranging from 2.5 to 30 mm in length (excluding antennae). The body is slightly flattened dorsoventrally, smooth or with a low dorsal keel (genus *Scina*) or armed with strong denticles and keels (genera *Ctenoscina*, *Spinoscina*, *Acanthoscina*), Amongst the Hyperiidea, this family is the richest in species. It is distributed throughout all regions of the world oceans from the Arctic Basin to the Antarctic. The Scinids are the most characteristic members of the mesopelagic, bathypelagic and abyssopelagic zones. Evidently, only a small number of species have secondarily adapted to living in surface waters while, at the same time, retaining their deepwater features (Vinogradov et al. (1996).

Many studies have examined the group of Hyperiidea (Zeidler 1990, Lima and Valentin 2001, Escobar-Briones et al. 2002, Shih and Hendrycks 2003, Zeidler 2004a, Zeidler 2004b, Brusca and Hendrickx 2005, Gasca et al. 2010, Zeidler 2015, Gasca and Hendrickx 2020). They are found in virtually any zooplankton and micronekton sample. Basic reference to this important group of crustaceans is the contribution by *Vinogradov et al. (1982)* and Vinogradov et al. (1996).

Numerous studies have been conducted to inventory pelagic Amphipoda and to document their distribution across different sectors of the Mediterranean Sea, including the works of Ruffo (Ruffo 1982, Ruffo 1989, Ruffo 1993), Stephensen (Stephensen 1918, Stephensen 1924, Stephensen 1925), *Chevreux (1919), Bakir et al. (2008)* and Bakir (2012), amongst many other historical and contemporary contributions. Despite this body of work, distributional data for Scinidae species within the Mediterranean remain relatively scarce and are represented by only a few references, such as LoBianco (1902), Laval (1980), Bakalem and Dauvin (1995), Vinogradov et al. (1996), Zelickman (2005), Ruffo (2010), Christodoulou et al. (2013) and Dauvin et al. (2025). Returning to local studies of Syrian coastal waters, only one species of Scinidae have been identified, which is *Scina borealis* (Hamameh 2014).

## Materials and methods

**Figure 1.**
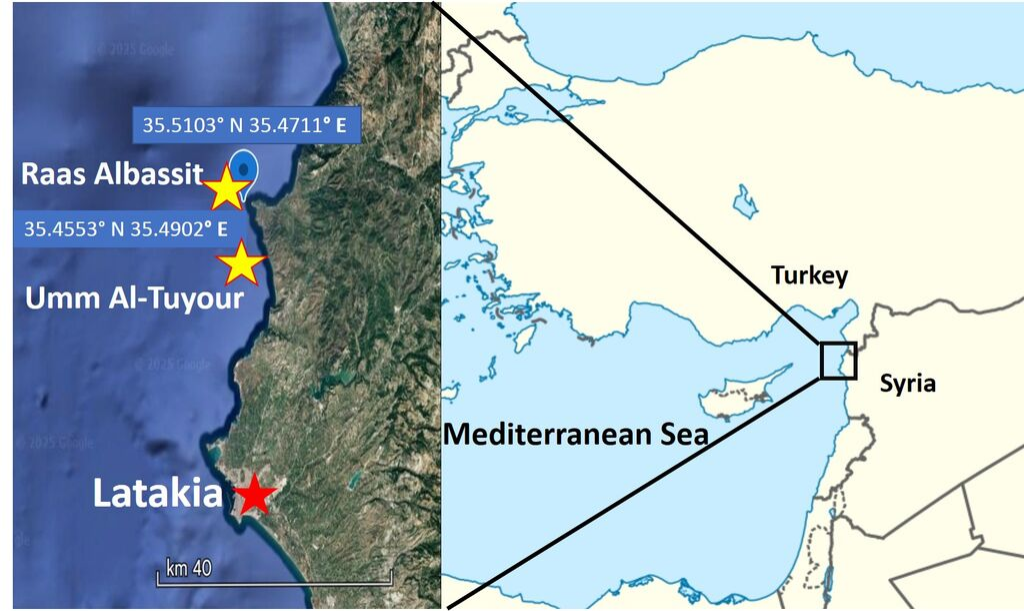
Sampling regions northern Latakia City, Syria, eastern Mediterranean (Google Earth).

Sampling was conducted seasonally between March and December 2024 (Table 1) in two regions north of Latakia, Syria: Ras Al Basit (35.5103° N, 35.4711° E) and Umm Al-Tuyour (35.4553° N, 35.4902° E), (Fig. 1).

Sampling was carried out on a regular schedule at a frequency of one offshore survey per month, during which one sample was collected from each designated depth. All samples were obtained from a single fixed station within each study area, located approximately 5 km offshore. Vertical hauls were performed at one station using a WP2 Closing Net with 200 µ mesh size, from six water layers to a depth of 500 m, (0-10, 10-25, 25-50, 50-100, 100-200, 200-500 m), with a total filtered water volume of ≈127.5 m^3^, corresponding to a filtration rate of ≈25.5 m^3^ per 100 m of vertical tow.

Immediately after collection, the samples were directly fixed using 4% formalin solution, ensuring the preservation of zooplankton organisms and the maintenance of their morphological integrity. Samples were stored in sealed 500 ml containers until they were transferred to the laboratory for detailed analysis.

Species identification was based on works by Wagler (1926), Zeidler (1990) and Vinogradov et al. (1996).

## Data resources

### Results

In this study, *Scina nana* was observed for the first time in the coastal waters of Syria (Latakia Basin), *S. nana* being recorded in July samples from 200-500 m, with low frequency of occurrence (three female), with the body length varying between 3.8 and 4.1 mm.

Global biodiversity repositories, including GBIF, OBIS and the authoritative taxonomic database WoRMS, consistently indicate that *Scina nana* is a globally rare holoplanktonic amphipod, represented by only a small number of confirmed occurrence records, all originating from open-ocean tropical and subtropical environments outside the Mediterranean Sea (GBIF 2026; OBIS 2026; WoRMS 2026). None of these databases contains any historical or contemporary evidence of the species within the Mediterranean Basin. This complete absence of records strongly suggests that the specimen documented from Syrian coastal waters represents the first known occurrence of *S. nana* in the eastern Mediterranean and, indeed, within the entire Basin.

The species *Scina similis* was also detected in the collected material; however, its occurrence was exceedingly rare, with only two females isolated throughout the study period. This species exhibits a high degree of morphological similarity to *S. nana*, making the distinction between the two taxa challenging and requiring careful anatomical examination. Despite its rarity, *S. similis* was successfully identified in the present study.

This species has been previously documented in the Mediterranean Sea, with confirmed records from central region of Mediterranean (LoBianco 1902), Stephensen (1918), Stephensen (1924) and Stephensen (1925) recorded it from near Sicily. Zelickman (2005) also lists *Scina similis* in the Mediterranean. The current finding represents the first documented occurrence of *S. similis* in the coastal waters of Syria, in the eastern Mediterranean, thereby extending its known geographical distribution and contributing a new record to the regional biodiversity.

The morphological characteristics of the fifth Pereopod (PV) have generally been relied upon to differentiate and classify this species and the schematic anatomical structure of the pereopods (V, VI, VII) for *Scina nana* are presented in Fig. 4, Fig. 5 and Figs 6, 7 for *Scina similis*.

## Taxon treatments

### Scina nana

Wagler, 1926

084930F3-75E4-5845-A4D1-31516577A1E4

https://www.gbif.org/occurrence/map?taxon_key=2217401

https://eol.org/pages/46521483

https://www.marinespecies.org/amphipoda/aphia.php?p=taxdetails&id=325398

Scina nana is described in Wagler (1926) and re-described in Vinogradov et al. (1996), p. 205 fig. 83.

#### Materials

- **Type status:** Other material. **Occurrence:** recordedBy: Hasan, B.H; individualCount: 3; **Taxon:** scientificName: *Scina nana*; originalNameUsage: *Scina nana* Wagler, 1926; order: Amphipoda; family: Scinidae; genus: *Scina*; specificEpithet: *nana*; scientificNameAuthorship: Wagler, 1926; **Location:** locationID: Latakia; waterBody: Mediterranean Sea; country: Syria; verbatimDepth: 200-500; minimumDepthInMeters: 200; maximumDepthInMeters: 500; verbatimCoordinates: 35.5103° N, 35.4711° E, and 35.4553° N, 35.4902° E; verbatimCoordinateSystem: 35.5103° N, 35.4711° E, and 35.4553° N, 35.4902° E; **Event:** eventDate: 2024 July; **Record Level:** basisOfRecord: PreservedSpecimen

#### Description

**Figure 2.**
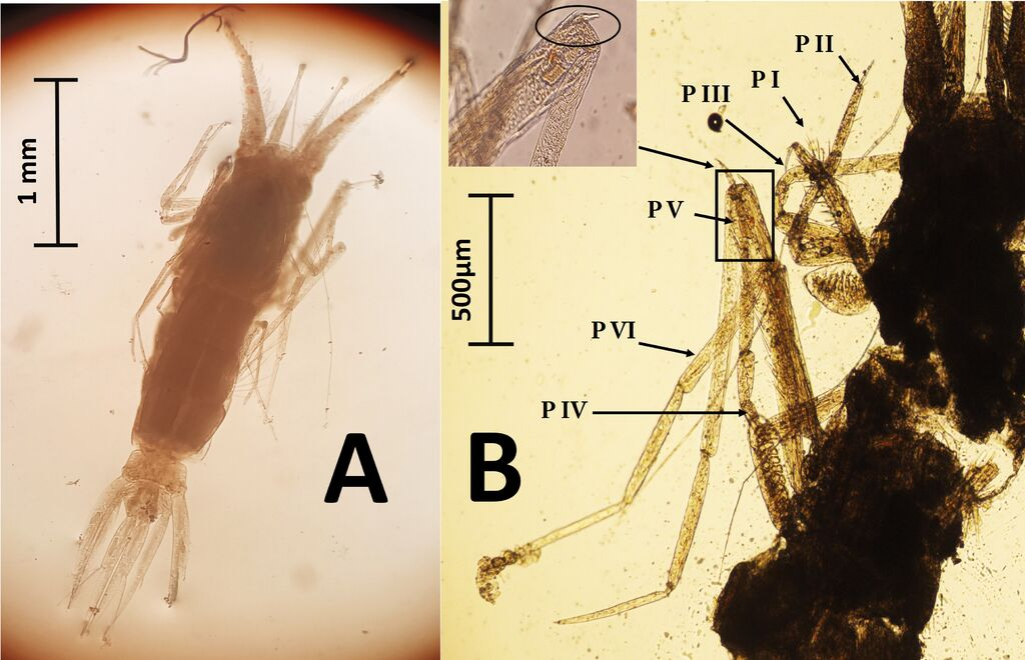
*Scina nana*: Female; **A** body (whole mount); **B** pereopods (I-VII) and the anterior margin of Pereopod (V) with two large denticles.

**Figure 3.**
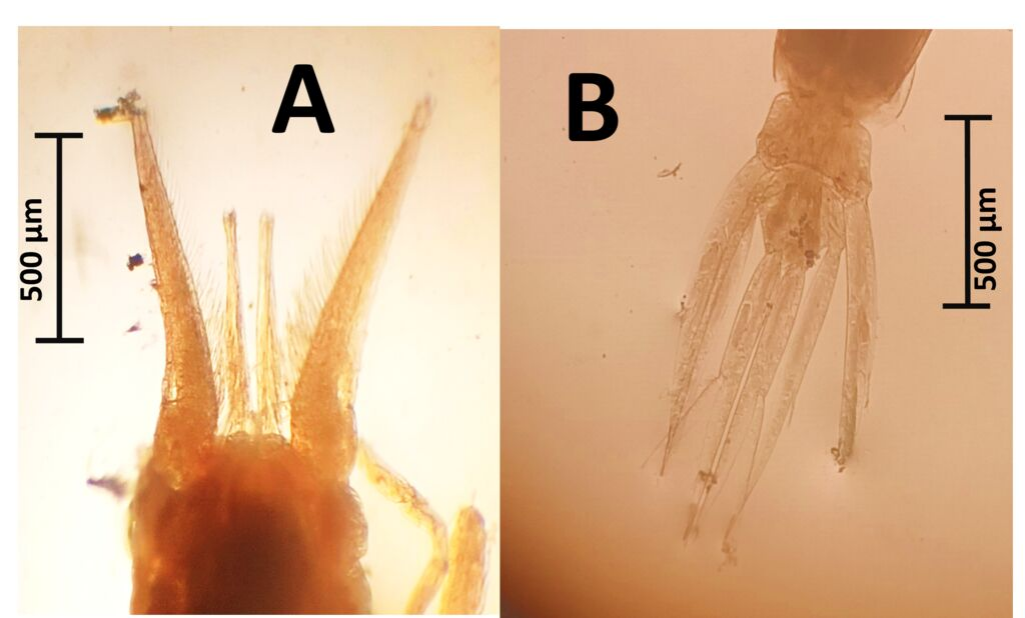
*Scina nana*: Female; **A** Head; **B** Urosome.

**Figure 4.**
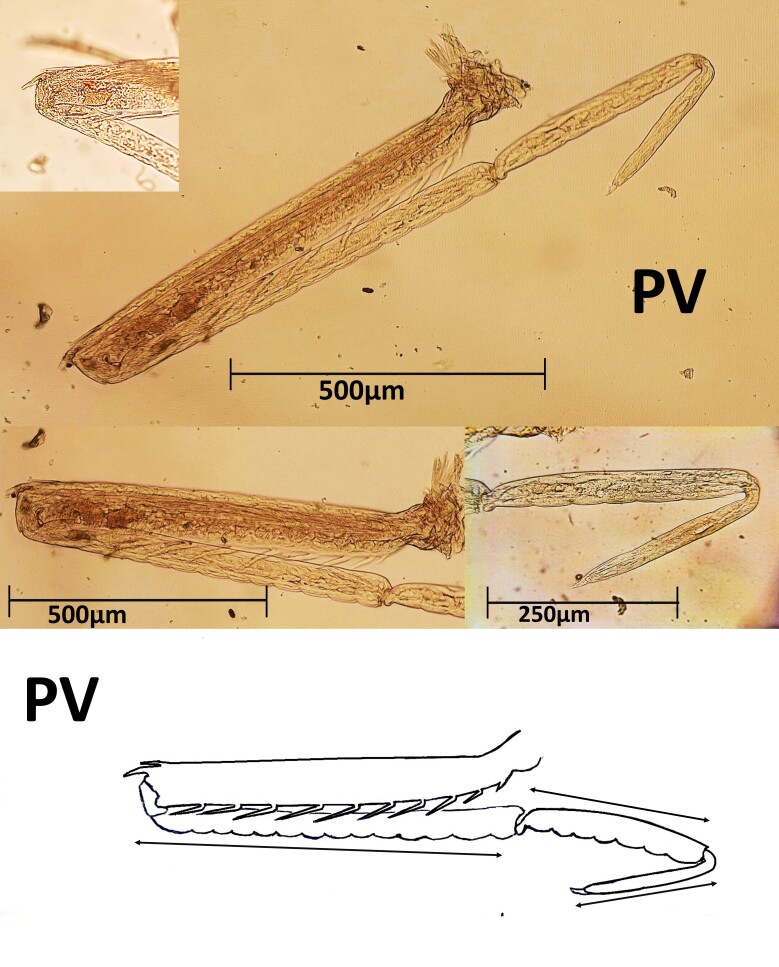
*Scina nana*: Female; pereopod (PV).

*Scina nana*: Length of sexually mature specimens 2-4 mm. The body is smooth and without keels. Antennae I are strong, equal in length to the pereon or slightly shorter than it. The second segment of pereopods V is armed on the posterior margin with long curved denticles, the anterior margin of the segment is smooth, with one or two large denticles only at the base of the short distal process, the fourth segment is slightly shorter than the second and roughly twice longer than the fifth, which in turn is somewhat longer than the sixth segment, the claw is very small (Vinogradov et al. 1996), (see Figs 2, 3, 4).

**Figure 5.**
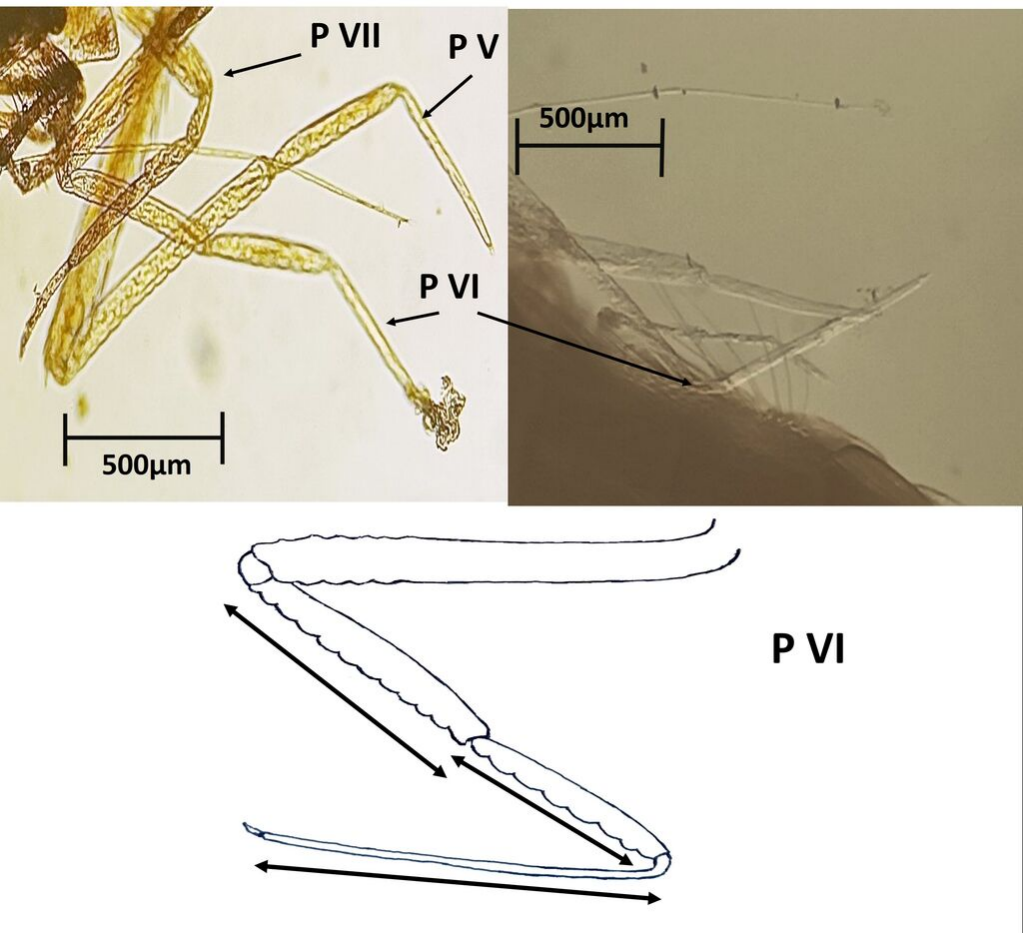
*Scina nana*: Female; pereopod (PVI); pereopod (PV, PVII).

**Figure 6.**
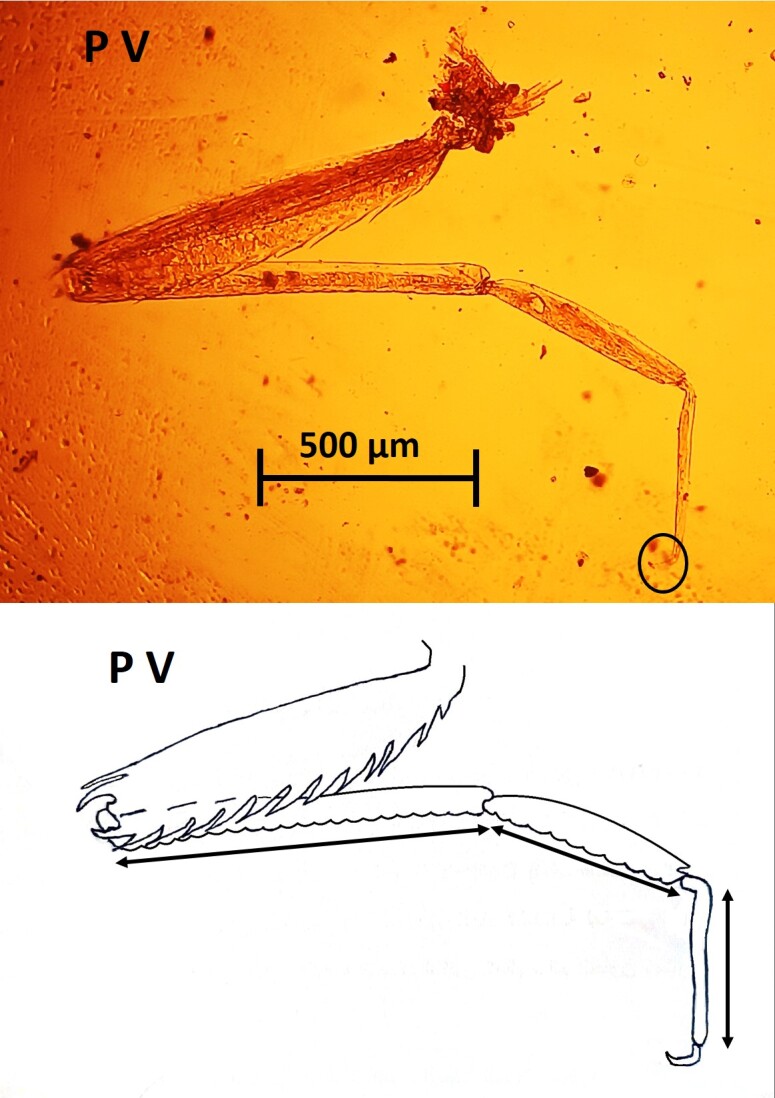
*Scina similis*: Female; pereopod (PV).

**Figure 7.**
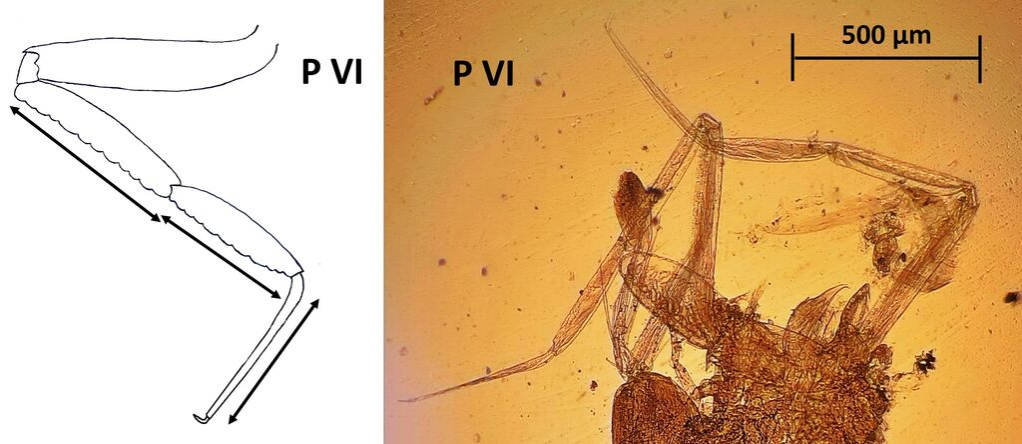
*Scina similis*: Female; pereopod (PVI).

The distinction between this species and closely-related forms *Scina similis* was established, based on the detailed morphological characteristics of pereopods (PV, PVI), (Figs 4, 5, 6, 7). In light of the observations made during the taxonomic examination, the configuration of terminal segments, the pattern of denticles and the number and arrangement of marginal serration, are summarised and comparatively presented in Table 2.

#### Distribution

*Scina nana* is relatively uncommon, found mainly in tropical regions of all the world’s oceans, except the Mediterranean Sea (Vinogradov et al. 1996, Zeidler and De Broyer 2009). In the Atlantic, it is known from the equatorial regions to about 33°S (02°36’N, 03°27’E), 200-0 m and (33°23’S, 16°19’E), 2000-0 m) (Zeidler and De Broyer 2009). In the Indian Ocean, off the West Ice Shelf (64°29’S, 85°27’E), (04°05’S, 73°24’E), 200-0 m, (02°38’S, 65°59’E), 2500-0 m, (03°24’S, 58°38’E), 2000-0 m, (03°26’S, 58°34’E), 1500-0 and (04°34’S, 53°42’E), 2000-0 m (Wagler 1927), it has been recorded only from equatorial regions. In the Pacific, it has been recorded only from the warmer waters of the Indo-Pacific/South China Sea region and off the Californian coast. It seems to inhabit near-surface waters (100-500 m), but has been found in catches from depths exceeding 2000 m to the surface (Zeidler and De Broyer 2009).

#### Notes

The specimen described in this study corresponds to the morphology of *Scina nana* described by Vinogradov et al. (1996) and available also in the original description by Wagler (1926).

## Discussion

In this study, *Scina nana* was observed for the first time in the coastal waters of Syria. This species is typically associated with tropical biogeographic layers at depths exceeding 200 m (Vinogradov et al. 1996, Zeidler and De Broyer 2009).

Globally, available data (GBIF, OBIS) show that *S. nana* is typically associated with deep-water layers exceeding 200 m, a distributional pattern consistent with the ecological characteristics of species belonging to genus *Scina*, which form part of the deep-pelagic holoplanktonic community. This pattern supports the interpretation of the species' presence in Syrian waters, as the depths at which it was recorded in the present study align with its known global ecological range.

Given the complete lack of previous Mediterranean records, the appearance of *S. nana* in the region is most plausibly interpreted as a recent event, supporting its classification as a non-indigenous species that has only recently entered the Basin. The rapid warming of Mediterranean waters in recent decades may have provided conditions favourable for the arrival or temporary establishment of this species. Additional factors such as broader changes in the thermohaline structure of the eastern Mediterranean may also have facilitated its introduction to the Syrian coast.

This record represents a signification addition to the biogeographical knowledge of holoplanktonic amphipods in the eastern Mediterranean and highlights the need for enhanced long-term monitoring programmes aimed at detecting their potential for establishment within the rapidly changing Mediterranean ecosystem.

## Supplementary Material

XML Treatment for Scina nana

## Figures and Tables

**Table 1.** Monthly sampling dates from the Syrian coastal waters.

**Month**	**Date**
March	14/3/2024
April	20/4/2024
May	18/5/2024
June	15/6/2024
July	13/7/2024
August	23/8/2024
September	14/9/2024
October	17/10/2024
November	23/11/2024
December	4/12/2024

**Table 2.** Comparison of diagnostic features and pereopods (PV, PVI) characteristics for *Scina nana* and *Scina similis*.

***S. similis***	***S. nana***	**Morphological Character**
Broad and nearly fusiform; the anterior margin ending with two denticles (one slender, second broader); the posterior margin with 13-14 curved denticles arranged in continuous series, with no distinct basal separation, giving the appearance that margin itself continues as series of denticles (Fig. 6)	Rectangular; the anterior and posterior margins nearly parallel; the anterior margin ending with two denticles (one slender, second broader); the posterior margin with 10 denticles that emerge strongly curved with a narrow base (Fig. 4)	Fifth pereopods_ Second segment (PV_ S2)
Slightly longer than second segment; the posterior margin smooth; the anterior margin with indentations forming more than 20 serrations (Fig. 6)	Shorter than second segment; the posterior margin smooth; the anterior margin with indentations forming 13-14 serrations (Fig. 4)	Fifth pereopods_ fourth segment (PV_ S4)
About half the length of fourth segment or slightly shorter; the anterior margin with 11-12 serrations (Fig. 6)	Slightly longer than sixth segment; the posterior margin smooth; the anterior margin with 6-7 serrations (Fig. 4)	Fifth pereopods_ fifth segment (PV_ S5)
Slightly shorter than fifth segment; ends in a long, curved claw (Fig. 6)	Ends in a short, nearly triangular claw (Fig. 4)	Fifth pereopods_ sixth segment (PV_ S6)
Matches Vinogradov et al. (1996) description being shorter than PV; the fourth segment is considerably shorter than the second segment; the fifth segment is somewhat shorter than the fourth segment; the sixth segment longer than the fifth and roughly equal to the fourth segment; the sixth segment ending in a long, curved claw; fourth segment with 11-12 serrations and fifth segment with 10-11 serrations (Fig. 7)	Matches Vinogradov et al. (1996) description being somewhat shorter than PV; the second segment is equal to the fourth and fifth together; the thin sixth segment is slightly longer than the fourth, but considerably longer than the fifth segment; the sixth segment very long ending in very small claw; the fourth segment with 11-12 serrations and fifth segment with 8-9 serrations (Fig. 5)	sixth pereopods_ General structure (PVI)
